# On the roles of function and selection in evolving systems

**DOI:** 10.1073/pnas.2310223120

**Published:** 2023-10-16

**Authors:** Michael L. Wong, Carol E. Cleland, Daniel Arend, Stuart Bartlett, H. James Cleaves, Heather Demarest, Anirudh Prabhu, Jonathan I. Lunine, Robert M. Hazen

**Affiliations:** ^a^Earth and Planets Laboratory, Carnegie Institution for Science, Washington, DC 20015; ^b^Sagan Fellow, NASA Hubble Fellowship Program, Space Telescope Science Institute, Baltimore, MD 21218; ^c^Department of Philosophy, University of Colorado, Boulder, CO 80309; ^d^Division of Geological and Planetary Sciences, California Institute of Technology, Pasadena, CA 91125; ^e^Earth Life Science Institute, Tokyo Institute of Technology, Tokyo 152-8550, Japan; ^f^Blue Marble Space Institute for Science, Seattle, WA 98104; ^g^Department of Astronomy, Cornell University, Ithaca, NY 14853

**Keywords:** selection, natural laws, evolving systems, functional information, Titan

## Abstract

Physical laws—such as the laws of motion, gravity, electromagnetism, and thermodynamics—codify the general behavior of varied macroscopic natural systems across space and time. We propose that an additional, hitherto-unarticulated law is required to characterize familiar macroscopic phenomena of our complex, evolving universe. An important feature of the classical laws of physics is the conceptual equivalence of specific characteristics shared by an extensive, seemingly diverse body of natural phenomena. Identifying potential equivalencies among disparate phenomena—for example, falling apples and orbiting moons or hot objects and compressed springs—has been instrumental in advancing the scientific understanding of our world through the articulation of laws of nature. A pervasive wonder of the natural world is the evolution of varied systems, including stars, minerals, atmospheres, and life. These evolving systems appear to be conceptually equivalent in that they display three notable attributes: 1) They form from numerous components that have the potential to adopt combinatorially vast numbers of different configurations; 2) processes exist that generate numerous different configurations; and 3) configurations are preferentially selected based on function. We identify universal concepts of selection—static persistence, dynamic persistence, and novelty generation—that underpin function and drive systems to evolve through the exchange of information between the environment and the system. Accordingly, we propose a “law of increasing functional information”: The functional information of a system will increase (i.e., the system will evolve) if many different configurations of the system undergo selection for one or more functions.

## Significance

The universe is replete with complex evolving systems, but the existing macroscopic physical laws do not seem to adequately describe these systems. Recognizing that the identification of conceptual equivalencies among disparate phenomena were foundational to developing previous laws of nature, we approach a potential “missing law” by looking for equivalencies among evolving systems. We suggest that all evolving systems—including but not limited to life—are composed of diverse components that can combine into configurational states that are then selected for or against based on function. We then identify the fundamental sources of selection—static persistence, dynamic persistence, and novelty generation—and propose a time-asymmetric law that states that the functional information of a system will increase over time when subjected to selection for function(s).

The laws of classical physics emerged as efforts to provide comprehensive, predictive explanations of phenomena in the macroscopic world (1–4). Well-documented examples include 10 statements that constitute classical laws of motion, gravity, electromagnetism, and energy (Table 1). Each of these empirically based natural laws describes a universal behavior of macroscopic physical systems.

**Table 1.** Ten laws of classical physics, adapted from refs. 3 and 4

Newton’s laws of motion	1. *First law of motion*: A body will continue in its state of rest, or of uniform motion in a straight line, unless it is compelled to change that state by forces imposed upon it. 2. *Second law of motion*: Force equals mass times acceleration. 3. *Third law of motion*: To every action force there is an equal and opposite reaction force.
Newton’s law of gravitational attraction	4. Between any two objects there is an attractive force proportional to the products of the two masses divided by the square of the distance between them.
Laws of thermodynamics	5. *First law of thermodynamics*: In a closed system, the total amount of energy is conserved. 6. *Second law of thermodynamics*: Heat will not flow spontaneously from a colder to a warmer body.
Laws of electromagnetism (electricity and magnetism are two aspects of the same force)	7. *Electrostatics (Coulomb’s Law)*: The force between any two electrically charged objects is proportional to the product of their charges divided by the square of the distance between them. 8. *Magnetostatics*: Every magnet is a dipole; like magnetic poles repel each other while unlike poles attract. 9. *Electromagnetism*: Magnetic fields are created by moving electrical charges. 10. *Electromagnetic induction*: Electrical fields and electrical currents are created by changing magnetic fields.

The list of laws in Table 1 is not meant to be exhaustive; for example, one might argue for the inclusion of additional conservation laws (e.g., for mass and charge). Nevertheless, collectively, these statements codify most phenomena of the natural world at scales of space and time that are perceptible to humans (i.e., nonrelativistic phenomena at scales greater than quantum effects). Note that while most of these natural laws can be quantified in the form of an equation, such a formulation is not an essential feature. The second law of thermodynamics, for example, is often presented as an inequality: The entropy of a closed system remains constant or increases (a statement that subsumes the observed asymmetry that “heat will not flow spontaneously from a colder to a warmer body”). Nevertheless, each of these laws is grounded in measurable parameters—mass, force, acceleration, distance, energy, or charge.

A rich variety of observable natural phenomena are explained by the classical laws of physics. The elliptical orbits of planets, the behavior of steam engines, the splitting of white light into a spectrum of colors by a prism, and the movement of a compass needle placed next to an electrical current all follow directly from these statements. The laws presented in Table 1 are some of the most important statements scientists have discovered to date, and while there may be disagreement about what would be included in a definitive list, conspicuously absent is a law of increasing “complexity.” Therefore, an important unanswered question is whether the thus-far-articulated natural laws, in combination with necessary background assumptions and initial conditions, are sufficient to codify all readily observed natural phenomena. Might additional natural laws of similar scope and import to those in Table 1 remain to be articulated (5–7)? If so, what steps must be taken to identify and codify such “missing laws”?

We propose an approach based on identifying multiple examples of disparate, ostensibly evolving macroscopic phenomena that exhibit striking conceptual similarities, strongly suggesting the possibility of an underlying, law-governed conceptual equivalence (*Natural Laws and the Discovery of Conceptual Equivalencies* and *Identifying a Promising Conceptual Equivalence among Disparate Evolving Systems*). One unifying aspect of all evolving systems is selection, so next we explore the fundamental sources of selection and function (*The Origins of Selection and Function*). We then introduce functional information as a parameter around which to formulate a natural law (*Functional Information and the Evolution of Systems*) and propose the law of increasing functional information (*The Law of Increasing Functional Information*). We conclude by examining important implications of this natural law (*Discussion*).

## Results

### Natural Laws and the Discovery of Conceptual Equivalencies.

The discovery of the classical laws of physics demonstrated that certain characteristics (i.e., properties or attributes) of physical bodies possess relevant similarities. While an apple’s fall and the Moon’s orbit are different in many ways, they are similar with respect to the apple and the Moon sharing the property of having mass, and thus the motions of both objects may be described in nonrelativistic contexts by Newton’s universal law of gravitational attraction. Sharing properties encompassed by a natural law serves to unify prima facie disparate phenomena at a conceptual level, suggesting that the phenomena concerned are equivalent in a theoretically important way and hence represent a unified domain of natural phenomena.

Establishing that a body of intuitively similar yet prima facie diverse phenomena are genuinely equivalent in a particular way depends upon the discovery of unifying, general laws subsuming them. For example, experiments performed by nineteenth-century physicists (e.g., Oersted and Faraday) led to the suspicion that electrical and magnetic phenomena were relevantly similar in important ways (8, 9). The conjectured conceptual equivalence between electricity and magnetism based on observations of both types of phenomena in laboratory settings was essential to developing the laws of electromagnetism, codified in Maxwell’s equations.

As another illustration, the discovery that heat can be converted to mechanical work (e.g., in steam engines) and that mechanical work can be converted to heat (e.g., through friction) suggested that hot objects and mechanical devices, such as a compressed spring, are conceptually equivalent insofar as they share the law-governed property of having energy (the dynamical property of having the capacity to do work). The concept of hot objects having energy led to the acceptance of the dynamical theory of heat and the kinetic theory of temperature (the idea that all matter is composed of particles constantly in motion, in which temperature is identified with mean kinetic energy, ½ *mv*^2^) over the now obsolete caloric theory (in which heat is transferred via a hypothetical, self-repellent, weightless fluid). Investigations into the efficiency of heat engines further revealed that the conversion of heat to mechanical work is not fully reversible—that heat flows spontaneously in only one direction, from a hot to a cold reservoir, which paved the way to the concept of entropy and the temporally asymmetric second law of thermodynamics. These investigations into the behavior of thermodynamic systems gave rise to the universal laws of thermodynamics, which describe relations among work, heat, and energy in material systems at or near equilibrium.

In sum, the discovery of the natural laws in Table 1 often began with a bold conjecture—namely, that seemingly unrelated phenomena are nonetheless similar in an important but as yet poorly understood way. The ability to articulate each of these theoretical laws critically depended upon identifying which among the conjectured theoretical attributes of the phenomena involved—e.g., inertial mass, energy, electrical charge—are lawfully related in an empirically revealing way. Empirical confirmation of the law established that the theoretical attributes concerned are “real” and indeed conceptually equivalent in this context.

Finally, it is important to keep in mind that even supposing that a body of natural phenomena are unifiable under an as-yet-unarticulated natural law, it does not follow that the set of attributes selected for formulating such a law will be successful. One’s ability to articulate empirically fruitful general laws of nature critically depends upon selecting the correct attributes for the phenomena concerned. For example, early theories of motion based on the now defunct theoretical concept of impetus (an internal motive property of objects keeping them in motion) were unable to account for important aspects of the motion of material objects and were replaced by Newton’s laws of motion, which were based on the very different theoretical property of inertia. In short, one can be wrong about which hypothesized theoretical characteristics are conceptually equivalent in the pertinent sense of providing a framework for unifying a body of prima facie disparate natural phenomena under a scientifically fruitful law.

The history of scientific discovery informs our search for empirically powerful, theoretical laws that describe the behavior of evolving systems considered generally—systems exhibiting a distinctive, temporally asymmetrical form of development—that is not obviously derivable from the classical laws of physics. Progress depends on examining a variety of evolving systems to identify potential conceptual equivalences through investigating common themes, perhaps subtle, that may point to a theoretical framework of basic attributes capable of supporting an as-yet-unarticulated law of nature unifying evolving phenomena.

### Identifying a Promising Conceptual Equivalence among Disparate Evolving Systems.

We define an “evolving system” as a collective phenomenon of many interacting components that displays a temporal increase in diversity, distribution, and patterned behavior. The concept of increased complexity is sometimes employed in this context (10–14). As such, evolving systems are a pervasive aspect of the natural world, occurring in numerous natural contexts at many spatial and temporal scales.

Evolving systems appear to display conceptual equivalences that are not as yet codified by any of the natural laws outlined in Table 1. Evolving systems must, according to our proposal, be consistent with all of the classical laws of physics even though their universal and distinctive behaviors are not adequately described by those laws, either singly or in combination. This requirement presents a potential challenge. The manifest tendency of evolving systems—especially nonliving ones, such as those involved in stellar, mineral, and atmospheric evolution (discussed below)—to become increasingly ordered with the passage of time seems to stand in contrast to the temporally asymmetrical character of the second law of thermodynamics, which characterizes natural phenomena as becoming increasingly disordered with the passage of time. One of the distinguishing features of our proposal is formulating a universal law for both living and nonliving evolving systems that is consistent with the second law of thermodynamics but may not follow inevitably from it.

What attributes might evolving systems have in common? In what ways might they be conceptually equivalent? Consider three examples of evolving systems.

1. Stellar evolution and nucleosynthesis: Stars begin as gravitationally bound masses of primarily hydrogen and helium in which internal pressures and temperatures are sufficiently high to initiate and sustain nuclear fusion reactions. The first stages of nucleosynthesis in all stars involve “hydrogen burning” to generate helium, but stars as large or larger than the Sun eventually undergo additional fusion processes: “helium burning” to produce carbon, “carbon burning” to form magnesium and other elements, and several successive stages that generate scores of elements and hundreds of isotopes (15–17). In the final, violent stages of stars’ lives, events such as classic novas, supernovas, and neutron star collisions generate the full periodic table of more than 100 elements and their ~2,000 isotopes. Thus, stellar evolution leads to new configurations of countless interacting nuclear particles. Inexorably, the system evolves from a small number of elements and isotopes to the diversity of atomic building blocks we see in the universe today.
2. Mineral evolution: “Mineral evolution” describes the changing diversity and distribution of minerals that arise during the formation and evolution of terrestrial planets and moons (18–21). The most ancient minerals in the cosmos include approximately 20 refractory phases that condensed from the expanding, cooling atmospheres of aged stars (22, 23). These minerals contribute to the dust and gas that form planets—materials that undergo further sequences of condensation, melting, crystallization, differentiation, alteration by temperature and pressure, and fluid-rock interactions (24). Each new physical, chemical, and (on Earth) biological process has the potential to diversify a planet’s mineral inventory. Thus, on Earth more than 5,900 mineral “species” have been codified (https://rruff.info/ima/; accessed 31 May 2023), with perhaps 3,500 additional species awaiting discovery and description (25). Furthermore, the average chemical and structural information contained in minerals increases systematically through billions of years of planetary evolution (26, 27). Thus, as with stellar nucleosynthesis, mineral evolution occurs as a sequence of processes that increase the system’s diversity in stages, each building on the ones that came before.
3. Biological evolution: Life is the quintessential evolving system. Charles Darwin (1859) proposed an elegant framework for life’s evolution by natural selection: 1) In any population, individuals display variations in their traits (due to, e.g., mutations and genetic recombination); 2) more individuals are born than can survive; and 3) individuals with heritable traits that promote survival are more likely to produce offspring that have those advantageous traits. Therefore, populations evolve through many generations as more advantageous traits are selected in preference to traits that do not confer advantage. Life, though distinct in the specifics of its evolutionary mechanisms, can be conceptualized as equivalent to the previous examples of nucleosynthesis and mineral evolution in the following way: Whether viewed at the scale of interacting molecules, cells, individuals, or ecosystems, biological systems have the potential to occur in numerous configurations, many different configurations are generated, and natural selection preferentially retains configurations with effective functions.

These three evolving natural systems differ significantly in detail. Stellar nucleosynthesis depends on the selection of stable configurations of protons and neutrons. Mineral evolution relies on selection of new, locally stable arrangements of chemical elements. Biological evolution occurs through natural selection of advantageous heritable traits. Nevertheless, we conjecture that these examples (and many others) are conceptually equivalent in three important respects (28, 29):

1. Each system is formed from numerous interacting units (e.g., nuclear particles, chemical elements, organic molecules, or cells) that result in combinatorially large numbers of possible configurations.
2. In each of these systems, ongoing processes generate large numbers of different configurations.
3. Some configurations, by virtue of their stability or other “competitive” advantage, are more likely to persist owing to selection for function.

In other words, each system evolves via the selection of advantageous configurations with respect to systemic persistence.

Ironically, these close parallels among disparate evolving systems may have been obscured by the power of Charles Darwin’s transformational arguments for biological evolution by natural selection (30). For example, the most vocal objections to the concept of mineral evolution are from biologists who argue that mineral systems cannot evolve—that evolution is a concept unique to biology (31). However, we contend that Darwinian natural selection and common descent are but one conceptually equivalent example of a far more general natural process (32). Details vary from system to system, but all evolving systems display the essential characteristics of combinatorial richness and selection for function.

Evolving systems are asymmetrical with respect to time; they display temporal increases in diversity, distribution, and/or patterned behavior. Previous work has attempted to describe this temporal asymmetry with a “theory of the adjacent possible,” a mathematical formula that describes a monotonic and explosive increase in combinatorial richness with time (33, 34). Nevertheless, evolution via selection is not constrained to be either continuous or permanent. Environmental circumstances that limit the generation of new configurations, or that restrict selection, will inhibit evolution. For example, nucleosynthesis halts when a star explodes, mineral evolution all but ceases if a planet freezes, the evolution of living systems may slow when a given environment exhibits little to no change over long timescales, and biological evolution is temporarily disrupted immediately following mass extinction events (although, in the long term, mass extinctions may open new niches for later evolutionary “explosions”). The fact that the rate of evolution varies when the mechanisms for sampling new configurations and/or a system’s selection pressures change highlights the meaningful connection between evolution and context.

These three characteristics—component diversity, configurational exploration, and selection—which we conjecture represent conceptual equivalences for all evolving natural systems, may be sufficient to articulate a qualitative law-like statement that is not implicit in the classical laws of physics. In all instances, evolution is a process by which configurations with a greater degree of function are preferentially selected, while nonfunctional configurations are winnowed out. We conclude:

Systems of many interacting agents display an increase in diversity, distribution, and/or patterned behavior when numerous configurations of the system are subject to selective pressure.

However, is there a universal basis for selection? And is there a more quantitative formalism underlying this conjectured conceptual equivalence—a formalism rooted in the transfer of information? We elaborate on these questions here and argue that the answer to both questions is yes.

### The Origins of Selection and Function.

The universe that we observe constantly generates certain ordered structures and patterned systems whose existence and change over time cannot adequately be explained by the hitherto identified laws of nature, such as those summarized in Table 1. The law-like statement we propose at the end of *Identifying a Promising Conceptual Equivalence among Disparate Evolving Systems* describes an increase in system complexity through the existence of selection pressures. In this section, we identify three kinds of selection and postulate how selective forces favor function.

#### Approaching first-order selection by imagining a patternless world.

A law of increasing complexity is by definition a temporally asymmetric law (i.e., the directionality of time is explicitly relevant to the changes in the state of the system). Modern physics explains the directionality of time by invoking the past hypothesis, which says that the universe began in an extremely low-entropy state (35, 36). The past hypothesis, in combination with the temporally symmetric fundamental laws of nature, results in the second law of thermodynamics, which states that total entropy tends to increase.

One strategy for identifying important aspects of our complexifying universe is to imagine a “possible world” with the same initial low-entropy state that marches through time in full accordance with the second law, but does not produce any systems of increasing complexity. What would be different about that world that prohibits order, diversity, and function to arise?

In this patternless world of our imagination, systems smoothly march toward states of higher entropy without generating any long-lived pockets of low entropy, for example, because of an absence of attractive forces (gravity, electrostatics) or universal overriding repulsive forces. That is, no barriers exist that prevent systems from taking a direct path to thermodynamic equilibrium as they evolve. It may not even be possible to draw boundaries between different macroscopic “entities” in such a universe: Can anything be distinguished if the entire universe is just a soup of matter and energy, quickly dissipating random fluctuations, and cooling off indefinitely?

Our universe is not that imaginary universe: It produces entities that do not take the most direct paths to their highest entropy states. Something “frustrates” and sometimes directs the dissipation of free energy, permitting the long-lived existence of disequilibria (37–41). What are some of these barriers? For nuclear fusion, Coulomb repulsion and the slow rate of weak-interaction events (which converts a proton into a neutron with the emission of a positron and an electron neutrino, creating a deuteron) prevent all of the hydrogen in the universe from spontaneously alchemizing into iron. For chemical reactions, kinetic barriers thwart many exergonic reactions, keeping chemical systems out of equilibrium. For heat transport, the low thermal conductivity of planetary material permits the persistence of thermal gradients inside planetary bodies.

The wide diversity of materials in our universe are a result of these barriers. The elements of the periodic table exist because light nuclei do not easily fuse to form iron, and many heavy nuclei are stable and do not decay. A visible photon does not by itself transform into many thermal photons. Minerals forged at the pressure and temperature conditions of Earth’s mantle can persist on the surface due to kinetic stability. Similarly, organic matter does not spontaneously combust in an oxygen atmosphere due to the high activation energy of combustion. We owe our existence to all of these metastable features of our universe.

Thus, the most basic selective force stems from the fundamental properties of our universe that allow for static persistence. (Such persistence is, of course, not absolutely indefinite but should extend over periods much greater than other time scales of local changes.) Many structures in nature have been selected for by their stability against decay to equilibrium. We can cast this as a “principle of static persistence,” which we call “first-order selection”:

Configurations of matter tend to persist unless kinetically favorable avenues exist for their incorporation into more stable configurations.

#### Core functions of dynamical systems and the origin of second-order selection.

Static persistence provides not only an enormous diversity of components, but it also provides “batteries of free energy” or “pockets of negentropy” throughout the universe that fuel dynamically persistent entities (42). Stars are dynamically persistent spheres of plasma that feed off the free energy liberated by nuclear fusion in their cores. Convection cells—be they inside a star or a planet’s core, mantle, or atmosphere—are dynamically persistent dissipative structures that feed off planetary thermal gradients. Life’s dynamical persistence is driven by chemical disequilibria in the environment and solar photons that are greatly out of equilibrium with the thermal background (39, 43, 44).

Dynamical entities are by necessity open systems. Therefore, unlike statically persistent entities, they are not defined by the persistence of a precise material composition: A star’s elemental abundances change over its lifetime; a hurricane incorporates many different parcels of air over its lifetime; organisms constantly exchange matter with their environment.

What, then, is persisting? In our view, it is processes, giving rise to what we call “second-order selection”:

Insofar as processes have causal efficacy over the internal state of a system or its external environment, they can be referred to as functions. If a function promotes the system’s persistence, it will be selected for.

The fundamental process is the dissipation of free energy—without this function, no complex, dynamic entities could exist. Unlike static persistence, which only requires dissipation during formation, dynamic persistence requires active dissipation. Other functions—such as autocatalysis, homeostasis, and information processing—can emerge that prolong the act of dissipation through space and time. For example, self-replicating systems—including life as we know it—are necessarily autocatalytic; all else being equal, variations of such systems that have greater autocatalytic prowess will propagate faster and can be characterized as having a higher “dynamic kinetic stability” (45, 46).

Different kinds of dynamical persistence are distinguished by the differing levels at which information plays a role in contributing to persistence. Here, information simply refers to patterns of data in a system that encodes about itself, its environment, or about its relation with its environment. In the case of autocatalysis, system information promotes persistence, whereas in homeostasis correlations between the system and its environment promote persistence. Information processing adds a dimension, as the system actively records information about the environment that promotes persistence. Note that information processing can occur at various degrees. Consider the differences between systems capable of memory, memory-based prediction, and prediction outside of memory. Memory (storage of information acquired through measurement and sensing) allows for encoding associations. Memory-based prediction (the ability to infer future states based on encoded memory) promotes persistence through basic causal understanding. Finally, prediction outside of memory requires imagination and the ability to consider counterfactuals; such abstraction allows for greater novelty generation through the generation of hitherto nonexistent, imagined versions of reality (47). This information-centric account of persistence and self-organization differs significantly from purely thermodynamic descriptions of “dissipative structures” (42, 48) and the physicochemical concept of dynamic kinetic stability (45, 46).

Let us call dissipation, autocatalysis, homeostasis, and information processing the “core functions” (49). Each of these functions serves to perpetuate itself by enabling further dissipation: Stars achieve homeostasis by balancing gravitational collapse with the kinetic energy generated by fusion, allowing fusion to persist; fire achieves autocatalysis by heating surrounding materials to combustion temperatures, prolonging burning; life achieves information processing through various learning mechanisms, including Darwinian evolution and neurological cognition, which in turn sustains the lineage of information transfer by promoting survival, propagation, and continued metabolic activity.

#### Ancillary functions, novelty search, and a third-order selection for novelty.

The most complicated systems may be nested networks of smaller complex systems, each persisting and helping to maintain the persistence of the whole. In nested complex systems, ancillary functions may arise. For example, enzymes are selected for their ability to catalyze a specific reaction, which may be one chemical transformation in an elaborate autocatalytic network, a homeostatic feedback system, or an information-processing apparatus. In other words, an enzyme’s function is not to perform any of the core functions alone, but to play a specific role in the context of a core function expressed at a higher level of organization. From the perspective of the enzyme, there is a top–down selection pressure for enzymes to have high catalytic efficiencies due to a selection pressure at a higher level for a lineage of organisms to persist. In other words, the enzyme’s function is informed by its context within a larger system. [Note one minor caveat: There are certain cases where protein folding is better described by selection for “form” based on the givens of physics—i.e., static persistence—rather than selection for functional adaptations (50)]. Ancillary functions can exist across many scales: From the perspective of an organism, there may be top–down selection pressures from the needs of its community, and the community may experience pressures from higher ecological units of selection, etc.

Ancillary functions may become so distant from core functions that it is difficult to understand their connections to the survival of the larger system. For example, the creation of art and music may seem to have very little to do with the maintenance of society, but their origins may stem from the need to transmit information and create bonds among communities, and to this day, they enrich life in innumerable ways. Perhaps, like eddies swirling off of a primary flow field, selection pressures for ancillary functions can become so distant from the core functions of their host systems that they can effectively be treated as independently evolving systems, perhaps eventually generating their own core functions [consider, for example, the elaborate dance culture that has been sexually selected for by generations of birds of paradise in Papua New Guinea (51)].

The ability to continually create (or discover?) new functions is a hallmark of life (52–56). Although some of these functions may seem neutral or even detrimental with regard to the stability of the whole system, overall the generation of novelty has the potential to further intertwine the core functions within a nest of feedback loops that supplement their stability and/or amplify their effectiveness. As a simplistic example, the invention of flight allowed animals new vectors by which to continue performing their core functions, making multiple lineages of organisms more successful at surviving and reproducing.

Another key feature of ancillary functions in biology is exaptation—a change in function over time (57). Returning to the example of flight, it has been suggested that insect wings initially served thermoregulatory purposes (58), and feathers may have performed thermoregulatory, display, and biomechanical support functions before aiding in flight (59). The concept of exaptation highlights the importance of context in the evolution of ancillary functions. Although thermoregulatory innovations tend to produce high-surface-area morphologies that can be flapped to generate cooling air currents, these structures will not be exapted for flight if they evolve in animals that are simply not predisposed to that manner of locomotion [consider, for example, the large ears of elephants (60)].

Adding new functions that promote the persistence of the core functions essentially raises a dynamic system’s “kinetic barrier” against decay toward equilibrium. Moreover, a system that can explore new portions of phase space may be able to access new sources of free energy that will help maintain the system out of equilibrium or move it even further from equilibrium. In general, in a universe that supports a vast possibility space of combinatorial richness, the discovery of new functional configurations is selected for when there are considerable numbers of functional configurations that have not yet been subjected to selection. Hence, we identify a “third-order selection” for novelty:

There exist selection pressures favoring systems that can open-endedly invent new functions—i.e., selection pressures for novelty generation.

The rise of art, literature, music, games, and technology in human culture may be reflections of our inherent desire to experiment with our world to discover new ways of thinking, being, and communing with one another. Although it may be argued that human-like innovation has negative adaptive value as evinced by flirtations with self-inflicted collapse (61), so far, our evolutionary “success” as a species may be attributed, in large part, to our curiosity (62). Perhaps it will be humanity’s ability to learn, invent, and adopt new collective modes of being that will lead to its long-term persistence as a planetary phenomenon (63–65). In light of these considerations, we suspect that the general principles of selection and function discussed here may also apply to the evolution of symbolic and social systems, but more detailed speculation is beyond the scope of the present paper.

The more interwoven a collection of systems becomes, the more ancillary functions emerge, and the more difficult it is to divine causal relationships independent from one another. In life, which is arguably the epitome of complex systems, causality is distributed across many different levels of organization, from the microscopic to the planetary. For instance, one’s emotions and actions are impacted by both gut microbiota (66–68) and by ethereal digital spaces that connect human minds across the globe (69–73). The prevailing model of life as a collection of well-defined individuals may need revision (74–80). We anticipate a biological paradigm shift analogous to the leap between classical mechanics and quantum mechanics: just as we replaced localized individual particles and discrete electron orbitals with wavefunctions and electron clouds, we may one day replace biological individuals with a “fuzzier,” networked picture of life. Such a view might still permit the existence of individual units but would stress the relationality among them in a process-based ontology (81, 82).

Finally, we can relate the three criteria underpinning all complex evolving systems (recall from *Identifying a Promising Conceptual Equivalence among Disparate Evolving Systems*: a diversity of components, a generative process for new combinations of components, and a selection pressure acting upon those combinations) to the concepts explored in this section. Component diversity across the universe is produced by inherent “kinetic” barriers that frustrate the immediate relaxation to equilibrium. These barriers also create opportunities for the synthesis of “batteries” of free energy that constitute the driving force for accessing different combinatorial states, which in general require thermodynamic work to create; for example, the onset of life is thought to be driven by redox disequilibria resulting from the evolution of nascent terrestrial planets (38, 39, 83–86). Configurations that are themselves statically persistent and promote dynamically persistent systems will be selected for.

### Functional Information and the Evolution of Systems.

All of the natural laws in Table 1 involve a quantitative parameter such as mass, energy, force, or acceleration. Is there an equivalent parameter associated with evolving systems? We suggest that the answer is information (measured in bits), specifically “functional information” as introduced by Szostak and coworkers (28, 87–89). Functional information quantifies the state of a system that can adopt numerous different configurations in terms of the information necessary to achieve a specified “degree of function,” where “function” may be as general as stability relative to other states or as specific as the efficiency of a particular enzymatic reaction.

Degree of function, *E_x_*, is a quantitative measure of a configuration’s ability to perform the function *x*. In an enzyme, for example, *E_x_* might be defined as the increase in a specific reaction rate that is achieved by the enzyme, whereas for a fluid flowing over a granular medium such as sand, where some form of periodic dune structure emerges, we could define *E_x_* as the minimum perturbation strength required to disrupt the dune structure. For a given set of parameters, the most persistent dune structure should be that which resists the largest range of perturbations (90, 91). The units of *E_x_* depend on the character of the function under consideration: The catalytic efficiency of an enzyme might be measured as a decrease in activation energy, for example, whereas the function of patterned sand might be to be maximally stable to external flow perturbations (28).

Functional information, *I*(*E_x_*), is calculated in terms of a specified degree of function (*E_x_*). In most cases, a minute fraction, *F*(*E_x_*), of all possible configurations of a system achieves a degree of function ≥ *E_x_.* Thus, functional information (in bits) is defined in terms of *F*(*E_x_*):

$$I\left(E_{x}\right)=-\log_{2}\left[F\left(E_{x}\right)\right].$$

In a system with *N* possible configurations (for example, an RNA sequence of *n* nucleotides with *N* = 4^n^ possible sequences, assuming equal probability for all sequences):

$$I\left(E_{x}\right)=-\log_{2}\left[M\left(E_{x}\right)/N\right],$$

where *M*(*E_x_*) equals the number of different RNA configurations with degree of function ≥ *E_x_*. Typically, the fraction of configurations, *F*(*E_x_*), capable of achieving a specified degree of function will decrease with increasing *E_x_* (28, 87).

This simple formalism leads to several important consequences. First, the greatest possible functional information for a given system occurs in the case of a single configuration that displays the highest possible degree of function, *E*_max_:

$$\begin{array}{c} I\left(E_{\max}\right)=-\log_{2}\left[1/N\right]=\log_{2}N\left(\mathrm{in}\;\mathrm{bits}\;\right). \end{array}$$

This maximum functional information is thus equivalent to the number of bits necessary and sufficient to specify any particular configuration of the system. On the other hand, the minimum functional information of any system is zero. This situation occurs for configurations with the lowest degree of function, *E*_min_, because all possible states have *E_x_* ≥ *E*_min_:

$$\begin{array}{c} I\left(E_{\min}\right)=-\log_{2}\left(N/N\;\right)=-\log_{2}\left(1\right)=0\;\mathrm{bits}. \end{array}$$

Thus, functional information must increase with degree of function, from zero for no function (or minimal function) to a maximum value corresponding to the number of bits that are both necessary and sufficient to specify any system configuration.

It is important to note that functional information is defined only in the context of a specific function *x*. For example, the functional information of every enzyme is greater than zero with respect to the catalysis of at least one specific reaction, but the functional information of that same enzyme is zero or minimal with respect to most other reactions. Functional information thus depends on both the system *and* on the context—the specific function under consideration. Note also that if no configuration of a system is able to accomplish a specific function *x* [i.e., if *M*(*E_x_*) = 0], then the functional information corresponding to that function is undefined, no matter how complex the patterning of that state appears to be.

Context specifies not only the relevant function and degree, *E*(*x*), but also the relevant facts about the system’s environment. We anticipate that our equation for functional information can be extended to include parameters specifying the environment. Such parameters would reflect the likelihood that a system achieves its degree of function, given different possible environmental states. Because these context-dependent features of the situation can be specified objectively, the context-dependence of functional information is not subjective.

The functional information formalism applies equally to both physical and symbolic systems (i.e., languages; computer code; scientific knowledge) because these evolving systems share three critical characteristics: 1) They consist of numerous interacting components, 2) the systems can occur in combinatorially large numbers of different configurations, and 3) selection processes favor some configurations that display useful functions.

A significant limitation of the functional information formalism is the difficulty in calculating *I*(*E_x_*) for most systems of interest. Functional information is a context-dependent statistical property of a system of many different agent configurations: *I*(*E_x_*) only has meaning with respect to each specific function. To quantify the functional information of any given configuration with respect to the function of interest, we need to know the distribution of *E_x_* for all possible system configurations relevant to the domain of interest. Determination of functional information, therefore, requires a comprehensive understanding of the system’s agents, their interactions, the diversity of configurations, and the resulting functions. Functional information analysis is thus not currently feasible for most complex evolving systems because of the combinatorial richness of configuration space. Even if we could analyze a specific instance where one configuration enables a function, we cannot generally know whether other solutions of equal or greater function might exist in configuration space (13).

### The Law of Increasing Functional Information.

The functional information formalism points to an important universal characteristic of evolving systems:

The functional information of a system will increase (i.e., the system will evolve) if many different configurations of the system are subjected to selection for one or more functions.

This proposition bears close parallels to the previously proposed “law of increasing complexity,” which states that natural selection, acting alone, tends to increase the complexity of a system (10, 11). The functional information formalism amplifies and quantifies this conjecture, which focuses on natural selection in evolving biological systems. We suggest that a law of functional information applies to a wide range of physical, biological, and symbolic systems: Any mechanism that selects from a population of states based on greater degrees of function will lead to increased functional information with respect to the selected function.

Why is this so? Let us explicitly address the temporal nature of functional information. Two things can cause the functional information of a system to increase over time: (a) The possibility space expands; or (b) the degree of function (i.e., the selection pressure) increases. Thus, a law of increasing functional information must not only rely upon 1) the existence of selection, but also 2) changes to the possibility space, and/or 3) changes in the selection pressure(s). In other words, *I*(*t*) responds to *F(E_x_*, *t*) and *E_x_*(*t*), so how and why do *F(E_x_*, *t*) and *E_x_*(*t*) change?

In *The Origins of Selection and Function*, we considered why selection for certain states and phenomena exists in our universe. Here, let us explore why the possibility space and selection pressures should change, first for statically persistent systems and then for dynamically persistent systems.

#### Increasing functional information in statically persistent systems.

Over time, new components become available as systems explore new pressure–temperature–composition (*P–T–X*) spaces. Cosmic cataclysms like supernovae and neutron star mergers expose stellar material to new *P–T–X* regimes where heavy nuclei are forged. When planets accrete from stardust and differentiate into immiscible layers, further *P–T–X* space is explored and novel minerals are created. Here, both (a) and (b) are occurring: The possibility space expands naturally as new components are invented in new *P–T–X* spaces, and the selection pressures change based on the *P–T–X* spaces that are explored. Thus, as time progresses, the functional information of statically persistent systems changes.

For example, in mineral evolution, we could attribute a rise in functional information to at least four factors: 1) the ever-increasing diversity and availability of elemental components in the universe; 2) the ever-increasing *P–T–X* spaces that perform mineral paragenesis; 3) the ever-increasing time between crystallization and the present during which minerals must resist alteration; and 4) the fact that new minerals often arise by modification of the prior generation of new minerals, leading to a kind of novelty that is only possible once the previous stage occurs.

#### Increasing functional information in dynamically persistent systems.

For dynamically persistent systems, the requirements for persistence are not simply set by inherent kinetic barriers to decay. The bar for persistence can change with time based on environmental factors, including other dynamic systems. In life, we often see coevolutionary “arms races” between species or groups, as exemplified by the “billions-year war” between viruses and cellular organisms (92). When two information processing systems compete, they can drive each other to higher and higher levels of sophistication and hence greater functional information via the generation of new ancillary functions (e.g., new forms of locomotion) or by reaching higher levels of information processing (e.g., the transition to consciousness) or new levels of organization (e.g., multicellularity). When new functions are added and/or the degree (*E_x_*) of those functions increases, the fraction of configuration space that can perform all of those functions grows smaller, and functional information increases. Thus, the ability of a system to respond to the selection pressure for novelty ensures that its functional information will increase.

One distinction with respect to life is the fact that biological evolution appears to be “open-ended,” forging adaptations and constructing new possibility spaces in an unpredictable and undecidable manner (55, 56, 93–95). In contrast, abiotic examples seem bounded. Recent work has estimated the combinatorial phase space of Earth’s present-day biosphere vastly outweighs the combinatorial phase space of the abiotic universe (96). Furthermore, biological and technological evolution seems to increase in its pace of innovation as a function of time (33, 97, 98). At the very least, life on Earth has evolved the ability to tune its evolvability (99–105).

It could be that life’s apparent open-endedness is merely due to the fact that the possibility space of living systems is far greater than that of abiotic systems. Such a large possibility space means that exponentially more time is necessary to explore it fully. Consequently, the fate of abiotic systems may appear deterministic because most of its possibility space will eventually be explored, while the fate of living systems appears open-ended because, over the same period of time, a smaller fraction of the possibility space will be explored. Indeed, some have argued that the universe is nonergodic above ~500 Da—that is, the universe has not had enough time to sample the entire combinatorial richness of complex organic molecules (106).

We postulate that an additional factor contributing to life’s apparent open-endedness is due to positive feedbacks in biological evolution that are absent in nonbiological systems—for example, as biological systems gain greater information-processing capabilities, they can learn to harness new forms of disequilibria, which can then fuel the discovery of novel learning mechanisms. This perspective is consistent with Chaisson’s observation of the increasing free energy rate density of complex systems in the universe (107); however, we suggest that this purely energetic account is incomplete. We propose a deep connection between free energy acquisition rate and information processing, unified by the principle of selection for core functions.

Stars (like the mineral example from *Increasing functional information in statically persistent systems*) go through successive stages—the triple-alpha process to make carbon must await sufficient hydrogen burning to make helium, etc.—so stars’ functional information could be said to increase with time. However, as dynamically persistent systems that do not partake in information processing, stars do not drive increases in their functional information in the same way that life does. The way that information cycles between life and its environment, and between life and itself, may therefore be life’s most distinguishing feature from other phenomena in the universe (108).

As a characteristic example of the coevolution between life and its environment, consider the Great Oxidation Event (GOE) (109). Although the details, timing, and history of the GOE are still debated (110–115), it is well accepted that the evolution of oxygenic photosynthesis in ancient Cyanobacteria pushed Earth’s atmospheric composition from an originally oxygen-poor state (<10^−5^ of the present atmospheric level) into a sequence of oxygen-rich states (currently 21% of the atmosphere). The GOE amplified all three drivers of complex evolving systems: 1) Abundant atmospheric O_2_ provided an extra source of component diversity (116); 2) O_2_ provided a new source of free energy to drive combinatorial exploration (117); and 3) as a highly reactive oxidant, O_2_ also provided a new set of selective criteria for persistence (118). Hence, the GOE is paradigmatic of how the Earth’s genesity—its ability to drive the evolution of complex systems (119)—and its functional information have increased over planetary history.

#### Application to an abiotic evolving chemical system: Titan.

As illustrated in the previous subsections, Earth history abounds with examples of increasing complexity that can be described via a law of increasing functional information. However, our proposed law should apply not only to the idiosyncrasies of Earth, but to a wide range of other planetary environments as well, in particular those for which life is not present. It is therefore informative to consider how the ideas outlined in this contribution are relevant to the highly chemically complex but very different environment of Saturn’s moon Titan. Titan is a planet-sized body with a dense N_2_–CH_4_ atmosphere that receives only enough solar radiation to maintain a surface temperature of 95 K at its equator. Liquid water—a key driver of mineralogical, geomorphological, and biological novelty on Earth—is therefore not stable on Titan’s surface. Methane, a liquid at the surface temperature of Titan and condensable in liquid and solid form in the atmosphere, plays some of the roles on Titan that water plays on Earth.

Titan is a prime astrobiological target because of its active stratospheric photochemistry, powered by ultraviolet (UV) light from the Sun, which produces a rich organic photochemistry including the production of organic aerosols that fall out onto a surface that is shielded from all but a small amount of cosmic (particle) radiation. Although there is no evidence that life exists on Titan, the moon’s rich organic chemistry teaches us much about how abiotic processes can generate chemical complexity and exhibit some degree of persistence-enhancing functionality.

For example, feedback loops among chemistry, radiation, and dynamics allow Titan’s current atmospheric state to persist on the order of ~3 × 10^7^ y (assuming no resupply of methane to the atmosphere) (120). Photochemically generated aerosols intercept incoming solar radiation in the middle of the atmosphere, creating a stratospheric thermal inversion, which inhibits convective motion. Higher-order hydrocarbons are thus trapped in a region of Titan’s atmosphere where they then amplify organosynthesis, producing further aerosol particles (121). Titan’s contemporary atmosphere is therefore in a state of dynamic persistence, where complex organic aerosols play a functional role in maintaining their own creation.

Titan’s atmosphere provides an example of a system in which there is rich component diversity (a mélange of C/H/N/O-bearing compounds) and a strong driving force for configurational exploration (UV photochemistry and energetic particles from cosmic rays), and some degree of selection for function (the autocatalytic feedback described above). However, selection for function in gas-phase chemistry in Titan’s atmosphere is weak because the primary products of the methane chemistry are orders of magnitude less volatile than methane itself and thus immediately condense into aerosols. The aerosols then grow and descend to altitudes below the UV-active zone on timescales of months to years (122). An interesting question is whether selection for function may be higher in these condensed phases because the chemistry occurs on surfaces rather than in the gas phase, reducing the degrees of freedom and enhancing reactivity. In what follows we consider chemistry once these materials have descended to Titan’s surface, where geological processes can act on them over a very wide range of timescales.

Most of the energy that reaches Titan’s surface is in the form of chemical bonds in the organic products of atmospheric methane chemistry, rather than photons (120), and much of this potential energy should be stored in the chemical bonds of acetylene (C_2_H_2_), based on photochemical models (121). At Titan’s surface, component diversity ought to be equivalent to that in the stratosphere, though the higher volatility of acetylene potentially allows it to concentrate toward the poles and away from organics with much lower vapor pressure such as hydrogen cyanide (HCN). Nonetheless, *Cassini* data show acetylene to be present at both low and high latitudes (123).

One possible outcome consistent with the law of increasing functional information is the formation of graphene from acetylene. Direct growth of graphene from acetylene on target substrates has been demonstrated (124), but growth may be favored if it increases the functionality of the overall organic system present on Titan’s surface in the following sense: Graphene is an excellent adsorber of molecular hydrogen (125). H_2_ is a principal product of the methane photolysis that is the starting point of all the photochemistry in Titan’s stratosphere. The vertical profile of H_2_ in Titan’s atmosphere (derived from measurements by the *Cassini* spacecraft and the *Huygens* lander) indicates a sink at the top, due to the escape of hydrogen to space, as expected, but also requires a sink at Titan’s surface, which was not predicted prior to *Cassini–Huygens* (126). Graphene provides a possible sink for molecular hydrogen (125), but this then begs the question of why graphene would be a favored product of further organic chemistry on Titan’s surface, versus other organic solids such as acetonitrile (C_2_H_3_N) or cyanoacetylene (C_3_HN).

Here, we propose that formation of graphene from acetylene could be the result of chemical evolution within which different possible configurations of the system are subject to selection for the function of adsorbing H_2_ as a waste product of photolysis. Formation of either acetonitrile or cyanoacetylene would be an evolutionary dead end because they are poor adsorbers of molecular hydrogen. But the formation of graphene permits large amounts of H_2_ to be trapped in a form available to reconstitute methane via reactions with the organic feedstock on Titan’s surface. Contact between the molecular hydrogen adsorbed in graphene and other organic molecules may occur by physical disturbances known to occur on Titan’s surface (methane rainstorms, fluvial transport, and winds), and energized by acetylene polymerization or the small but nonzero flux of particle radiation from high energy galactic cosmic rays (120).

Applying the considerations given above, the functional information of a surface environment where acetylene is converted to graphene on a suitable templating surface is greater than zero, but the functional information of a surface environment where acetylene forms simpler structures (such as cyanoacetylene) that do not require templating would be zero. In the former case, hydrogen can be stored, leading to further reactions in an otherwise hydrogen-poor local environment; in the latter, hydrogen would not be made available. Functional information thus depends, as noted above, on both the system (acetylene forming graphene on a suitable template) and on the specific function under consideration, namely molecular hydrogen storage. Typical templates for graphene formation are metallic, but a monolayer of preexisting graphene seems to allow growth of additional graphene layers (127). We can imagine growth occurring on a suitable metallic surface delivered stochastically by a large meteorite impact, providing the seed template for further production of graphene.

If hydrogen stored in graphene does react to produce methane, it would promote the persistence of Titan’s methane-rich atmosphere, which is the essential starting point for all Titan organosynthesis. This self-sustaining behavior of Titan’s hydrogen/methane cycle would be a strong test of the hypothesis that there is a law of increasing functional information operable in an abiotic organic system that has no access to or interaction with a biological system.

As stated in *Functional Information and the Evolution of Systems*, it is difficult to calculate the absolute value of the functional information of Titan’s atmosphere–surface system given the vastness of the total configuration space of such a system. However, the general scheme would proceed as follows: 1) decide how to describe the different configurations of the system (e.g., by the distributions of carbon and hydrogen atoms); 2) identify the relevant functions (e.g., cycling of methane via interactions with graphene); 3) calculate the functional information by determining the fraction of configuration space that can perform this function to the relevant degree (i.e., those configurations that maintain the dynamic persistence of a hydrocarbon-rich atmosphere). One prescription would be to count the number of configurations of carbon atoms that provide a high storage capacity for chemically available hydrogen, relative to the total configurations of carbon atoms in the various solids produced by methane photochemistry and subsequent surface chemistry. Since graphene has a very particular structure that allows for high uptake of hydrogen, *M*(*E_x_*)/*N* would be a small number and the functional information measure *I*(*E_x_*) large. Even without knowing the absolute value of the functional information of Titan’s atmosphere–surface system, if long-term dynamic persistence due to chemical evolution is at play on that world, it would indicate that the system’s functional information has increased over geologic time.

The existence of graphene on Titan’s surface in place of or in coexistence with simpler products of acetylene chemistry is speculative, but it may be determined with NASA’s *Dragonfly* mission (128). As a rotorcraft designed to sample various types of organic-rich surfaces on Titan with a mass spectrometer, the mission will be able to search for a broad suite of organic compounds indicative of chemical evolution on the surface, and thereby test the ideas presented here.

## Discussion

The law of increasing functional information has several implications for the behavior of evolving systems.

1. Some functions are inherently more information-rich than others: Some ribozymes, for example, require sequence lengths of several dozen nucleotides to display any significant degree of function. If, however, a ribozyme achieves a high degree of function with, for example, a sequence of only 10 nucleotides, then the functional information cannot exceed *I*_max_ ≤ −log_2_(1/4^10^) = 20 bits. Similarly, some concepts in science or mathematics can be fully expressed with fewer bits than others. In the context of life’s origin, this observation underscores the possibility that if some key functional prebiotic macromolecules require only a few monomers then their occurrence may be deterministic because all possible short sequences are likely to be present in a local population.
2. Functions that are not under selective pressure may remain constant or decrease in their degree of function: A number of researchers have demonstrated that in biological systems “length selection pressure” may result in a substantial reduction in the optimal system size, especially when limited resources or rapid replication confers an advantage on parsimonious genomes (12, 129–131). Such a reduction may involve the loss of some functions, yet still represent an increase in the functional information of the selected function.
3. Differing systems possess varying degrees to which they can continue to evolve: “Potential complexity” or “future complexity” have been proposed as metrics of how much more complex an evolving system might become (12). Functional information has the potential to facilitate the estimation of these parameters. If, for a given function, we can determine the relationship between *I*(*E_x_*) and *E_x_*, then we can deduce how close we are to *E*_max_ [and *I*(*E*_max_)]. We can then estimate the prospects of modifying parameters that might increase the functional information of the system.
4. The rate of evolution of some systems can be influenced artificially: The functional information formalism suggests that the rate of evolution in a system might be increased in at least three ways: 1) by increasing the number and/or diversity of interacting agents, 2) by increasing the number of different configurations of the system; and/or 3) by enhancing the selective pressure on the system (for example, in chemical systems by more frequent cycles of heating/cooling or wetting/drying). Future experiments in chemical evolution as well as simulations of artificial evolving systems (especially those involving novelty search) may be able to place quantitative measures on how the rate of evolution can be tuned by varying important parameters and driving forces.
5. Evolving systems are overlapping and interdependent: The examples of nucleosynthesis, minerals, and biology are but three examples of the deep connections among evolving systems. Minerals could not have formed without prior nucleosynthesis, while life (by most accounts) could not have emerged without minerals (132–138). Similarly, numerous evolving technological and symbolic systems had to await the evolution of human society (139). The fabric of information transfers inherent in these interwoven systems constitute a kind of pervasive “information field” with gradients and rates of transfer that may have parallels in physical and chemical systems.

Given the ubiquity of evolving systems in the natural world, it seems odd that one or more laws describing their behaviors have not been more quickly forthcoming [though note the important contribution of Price (140)]. Perhaps the dominance of Darwinian thinking—the false equating of biological natural selection to “evolution” writ large—played some role. Yet that cannot be the whole story.

A more deeply rooted factor in the absence of a law of evolution may be the reluctance of scientists to consider “function” and “context” in their formulations. A metric of information that is based on functionality suggests that considerations of the context of a system alters the outcome of a calculation, and that this context results in a preference for configurations with greater degrees of function. An asymmetric trajectory based upon functionality may seem antithetical to scientific analysis. Nevertheless, we conjecture that selection based on static persistence, dynamic persistence, and novelty generation is a universal process that results in systems with increased functional information.

## Data Availability

There are no data underlying this work.
